# 单孔胸腔镜手术联合ERAS理念指导下的呼吸功能锻炼在肺癌围手术期的应用

**DOI:** 10.3779/j.issn.1009-3419.2020.101.26

**Published:** 2020-08-20

**Authors:** 庆彤 施, 亚利 刁, 军 钱

**Affiliations:** 1 225000 扬州, 扬州大学附属医院胸外科 Department of Thoracic Surgery, the Affiliated Hospital of Yangzhou University, Yangzhou 225000, China; 2 224000 盐城, 盐城市第一人民医院胸外科 Department of Thoracic Surgery, Yancheng First People's Hospital, Yancheng 224000, China

**Keywords:** 肺肿瘤, 快速康复外科, 单孔胸腔镜手术, Lung neoplasms, Enhanced recovery after surgery, Single-hole thoracoscopic surgery

## Abstract

**背景和目的:**

肺癌是当前国内和国外发病率和死亡率均排名前列的恶性肿瘤, 手术治疗为肺癌治疗的主要治疗方案。本研究旨在探讨快速康复外科(enhanced recovery after surgery, ERAS)理念的呼吸功能锻炼联合单孔胸腔镜手术对肺癌患者术后肺部并发症、术后疼痛、下床时间、拔管时间以及住院时间的影响。

**方法:**

选择2017年10月-2019年10月在扬州大学附属医院和盐城市第一人民医院行胸腔镜肺癌手术的240例患者, 随机分为四组, 每组60例。A组患者采用单孔胸腔镜手术, 术前进行ERAS理念宣教和呼吸功能锻炼; B组采用常规3孔胸腔镜手术, 术前进行ERAS理念宣教和呼吸功能锻炼; C组采用常规3孔胸腔镜手术, 进行常规入院宣教和护理指导, 常规的呼吸功能锻炼, 无术前ERAS理念宣教; D组采用单孔胸腔镜手术, 进行常规入院宣教和护理指导以及常规的呼吸功能锻炼, 无术前ERAS理念宣教。记录四组患者术后肺部并发症的发生数量、术后疼痛、下次时间、拔管时间及住院时间。

**结果:**

A组分别与B组、C组、D组三组比较, 肺部并发症发生率明显降低, 下床时间、拔管时间和住院时间明显缩短; 与B组、C组两组比较, 术后疼痛明显减轻; B组与C组比较, B组肺部并发症显著降低, 下床时间、拔管时间和住院时间明显缩短, 差异均有统计学意义(*P* < 0.05)。术后疼痛在A组和D组比较以及B组和C组比较中均未见明显差异(*P* > 0.05)。

**结论:**

对于单孔胸腔镜肺癌手术患者, ERAS理念指导可有效降低肺部并发症的发生率和术后疼痛, 缩短下床时间、拔管时间以及住院时间。

## Abstract (en)

**Background and objective:**

Lung cancer is currently the leading malignant tumor in both domestic and foreign morbidity and mortality. Surgical treatment is the main treatment option for lung cancer. The aim of this study is to explore the effects of enhanced recovery after surgery (ERAS) combined with respiratory function exercise combined with single-hole thoracoscopic surgery on lung cancer patients with postoperative pulmonary complications, postoperative pain, time to get out of bed, time to extubation and length of hospital stay.

**Methods:**

A total of 240 patients who underwent endoscopic lung cancer surgery at the Affiliated Hospital of Yangzhou University and the Yancheng First People's Hospital from October 2017 to October 2019 were randomly divided into 4 groups, with 60 patients in each group. Patients in group A underwent single-hole thoracoscopic surgery, and preoperatively performed ERAS concept education and respiratory function training; group B used conventional 3-hole thoracoscopic surgery, and performed ERAS concept education and respiratory function training before operation; group C used conventional 3-hole thoracoscopic operation surgery, routine hospitalization education and nursing guidance, routine respiratory function training, no preoperative ERAS concept education; group D used single-hole thoracoscopic surgery, routine hospitalization education and nursing guidance, routine respiratory function training, no preoperative ERAS concept mission. The number of postoperative pulmonary complications, postoperative pain, time to get out of bed, extubation time, and hospital stay were recorded in the four groups.

**Results:**

Compared with the groups B, C, and D, the incidence of pulmonary complications was significantly reduced, and the time to get out of bed, extubation time, and hospital stay were significantly shortened in group A. Compared with groups B, C, the postoperative pain was significantly reduced in group A. Compared with group C, the pulmonary complications were significantly reduced, and the time to get out of bed, extubation time and hospital stay were significantly shortened in group B. The differences were statistically significant (*P* < 0.05). There was no significant difference in postoperative pain between group A and group D, group B and group C (*P* > 0.05).

**Conclusion:**

For patients with single-hole thoracoscopic lung cancer surgery, the ERAS concept guidance can effectively reduce the incidence of pulmonary complications and postoperative pain, shorten the time to get out of bed, the time to extubate, and the length of hospital stay.

随着空气质量的恶化, 室内装修污染的加重, 工作、生活压力的日益增加以及体检的逐渐普及, 肺癌尤其是早期肺癌已成为目前胸外科最常见的疾病, 2012年全球有182万新发病例和156万死亡病例^[1]^。

肺癌是我国目前死亡率最高的疾病, 早期治疗可以获得较好的远期生存率^[2]^。肺癌的治疗方式有手术治疗、免疫治疗以及靶向治疗等。手术方式目前有胸腔镜手术和开放手术, 随着手术器械的改进和手术技术的提高, 胸腔镜手术已成为了主要的手术方式。胸腔镜手术的方式分为单孔手术和多孔手术, 多孔手术里以3孔最为普遍^[3]^。快速康复外科(enhanced recovery after surgery, ERAS)自我国引进以来在多个学科都取得了较好的疗效, 但未得到广泛的推广, 扬州大学附属医院和盐城市第一人民医院胸外科在单孔胸腔镜手术治疗联合ERAS理念指导下的呼吸功能锻炼, 促进了患者的快速康复, 现总结如下。

## 1 资料与方法

### 1.1 一般资料

选取2017年10月-2019年10月扬州大学附属医院胸外科和盐城市第一人民医院收治的肺癌患者240例, 经医院伦理委员会批准, 使用区组随机分组方法, 以入院时间(日)作为配伍因素, 将入院时间同日相邻的10例患者作为一个区组, 分为A组、B组、C组、D组四组。A组采用单孔胸腔镜手术, 术前进行ERAS理念宣教、常规的呼吸功能锻炼和使用呼吸训练器进行呼吸功能锻炼; B组采用常规3孔胸腔镜手术, 术前进行ERAS理念宣教、常规的呼吸功能锻炼和使用呼吸训练器进行呼吸功能锻炼; C组采用常规3孔胸腔镜手术, 仅进行常规入院宣教和护理指导以及常规呼吸功能锻炼, 无术前ERAS理念宣教; D组采用单孔胸腔镜手术, 仅进行常规入院宣教和护理指导, 常规呼吸功能锻炼, 无术前ERAS理念宣教, 每组60例。(1)纳入标准：①术前胸部计算机断层扫描(computed tomography, CT)检查和术后病理结果诊断为肺癌; ②心、肺、肝、肾等重要脏器和凝血功能能耐受手术; ③行胸部CT、腹部CT、头颅磁共振成像(magnetic resonance imaging, MRI)、全身骨显像检查或正电子发射型计算机断层显像(positron emission tomography-CT, PET-CT)检查, 未发现有远处转移; ④术前未行放疗和化疗, 全身麻醉下行胸腔镜下肺叶切除+纵隔淋巴结清扫术; ⑤签署知情同意书。(2)排除标准：①肿瘤侵犯重要血管或重要脏器, 或出现远处转移, 无法行根治手术; ②既往有严重呼吸系统疾病史; ③术前行放疗或化疗; ④术后出现严重并发症; ⑤术后病理提示良性; ⑥依从性差。

### 1.2 方法

(1) 呼吸功能锻炼组, 包括A组和B组：①患者使用呼吸训练器进行吸气和呼气锻炼, 嘱患者处于站位或半卧位, 闭口用鼻子尽力吸气, 根据患者具体情况进行憋气(30 s-60 s), 而后缩唇轻闭慢慢进行呼气, 吸气和呼气的时间比例为1:2或者1:3, 要做到深吸慢呼, 缩唇程度以不感觉到费力为适度。呼吸功能训练需到达设定目标, 如不能则以吹不动或吸不动为止; 根据患者体重×10 mL/kg计算潮气量, 每日训练3次, 共训练1周; ②爬楼梯训练：爬楼时时采用缩唇呼吸, 膝盖保持轻度屈曲, 避免过度负重造成膝关节受伤, 爬楼至轻度气喘时停止, 每天两次, 约15 min-30 min^[4]^。(2)常规护理组：给予日常入院宣教, 包括戒烟、介绍管床医务人员以及病区环境、术前检查的注意事项、术前及术后的饮食注意事项。常规的呼吸功能锻炼：爬楼梯训练法。

### 1.3 观察指标

三组患者术后肺部并发症的发生情况, 包括肺部感染、肺不张和呼吸功能衰竭。(1)三组患者术后24 h、48 h和72 h疼痛情况; 术后疼痛情况评估使用数字评定量表(numeric rating scale, NRS)^[5]^, 术前宣教时告知使用方法。0分表示无痛, 1分-3分表示轻度疼痛, 4分-6分表示中度疼痛, 7分-9分表示重度疼痛, 10分表示剧烈疼痛。(2)三组患者的下床时间、拔管时间和住院时间。下床时间记录为患者下床活动 > 5 min的时间; 拔管时间为胸管引流量少于100 mL/24 h, 胸片检查无胸腔积液时拔管的时间; 住院时间为手术日至出院日之间的时间。

### 1.4 统计学方法

采用SPSS 25.0进行统计学分析, 计量资料与计数资料分别以(Mean±SD)及率(%)表示, 组间比较分别采用*t*及χ^2^检验, 以*P* < 0.05为差异有统计学意义。

## 2 结果

### 2.1 各组患者基线资料比较

A组、B组、C组、D组患者的性别构成、肿瘤的类型、平均年龄、手术部位和肿瘤分期的差异均无统计学意义(*P* > 0.05)(表 1)。

**1:** 四组患者的临床特征(*n*=60) Clinical characteristics of four groups (*n*=60)

Variables	Group A	Group B	Group C	Group D	*F*/*X*^2^	*P*
Age (Mean±SD, yr)	59.83±10.42	61.57±10.58	60.63±10.61	61.49±10.57	0.350	0.545
Gender					3.030	0.078
Male	33 (55.00%)	35 (58.33%)	38 (63.33%)	36 (60.00%)		
Female	27 (45.00%)	25 (41.67%)	22 (36.67%)	24 (40.00%)		
Location					3.430	0.487
Left upper lobe	13 (21.67%)	12 (20.0%)	15 (25.00%)	11 (18.33%)		
Left lower lobe	9 (15.00%)	8 (13.33%)	9 (15.00%)	7 (11.67%)		
Right upper lobe	20 (33.33%)	23 (38.33%)	19 (31.67%)	21 (35.00%)		
Right middle lobe	7 (11.67%)	6 (10.00%)	8 (13.33%)	9 (15.00%)		
Right lower lobe	11 (18.33%)	11 (18.33%)	9 (15.00%)	12 (20.00%)		
Pathological stage					0.380	0.773
Stage I	35 (58.33%)	38 (63.33%)	39 (65.00%)	40 (66.67%)		
Stage II	21 (35.00%)	19 (31.67%)	16 (26.67%)	18 (30.00%)		
Stage III	4 (6.67%)	3 (5.00%)	5 (8.33%)	2 (3.33%)		

### 2.2 三组患者术后肺部并发症比较

A组与B组、C组分别比较肺部并发症的发生率显著降低; B组与C组比较, 肺部并发症的发生率也明显降低, 差异有统计学意义(*P* < 0.05)(表 2)。

**2:** 三组患者术后肺部并发症比较 Comparison of postoperative pulmonary complications in four groups of patients

Pulmonary complications	Group A	Group B	Group C	Group D	*X*_1_^2^	*X*_2_^2^	*X*_3_^2^	*X*_4_^2^	*P_1_*	*P_2_*	*P_3_*	*P_4_*
Lung infection	3 (5.00%)	8 (13.33%)	15 (25.00%)	13 (21.67%)	8.532	10.645	6.293	10.086	0.022	0.011	0.031	0.014
Atelectasis	3 (5.00%)	6 (10.00%)	12 (20.00%)	11 (18.33%)	8.195	12.728	6.673	11.912	0.019	0.013	0.027	0.015
Respiratory failure	1 (1.67%)	3 (5.00%)	7 (11.67%)	6 (10.00%)					0.015	0.008	0.021	0.020
*X*_1_^2^、*P*_1_ is the comparison between groups A and B; *X*_2_^2^、*P*_2_ is the comparison between groups A and C; *X*_3_^2^、*P*_3_ is the comparison between groups B and C; *X*_4_^2^、*P*_4_ is the comparison between groups A and D.												

### 2.3 三组患者术后疼痛情况

三组术后1 h疼痛无明显差异。术后24 h、48 h和72 h, A组术后疼痛程度均较B组和C组明显降低, 差异有统计学意义(*P* < 0.05)。B组与C组术后疼痛程度基本相似, 无统计学差异(*P* > 0.05)(表 3)。

**3:** 三组疼痛评分和下床、拔管、出院时间比较(Mean±SD) Comparison of pain scores, time to get out of bed, extubation, and discharge from three groups (Mean±SD)

Index	Group A	Group B	Group C	Group D	*X*_1_^2^	*X*_2_^2^	*X*_3_^2^	*X*_4_^2^	*P_1_*	*P_2_*	*P_3_*	*P_4_*
Pain (NRS)												
1 h after operation	5.81±1.58	5.72±1.63	5.77±1.61	5.83±1.65	0.355	0.373	0.279	0.332	0.801	0.785	0.821	0.739
24 h after operation	6.31±1.38	7.35±1.41	7.43±1.35	6.38±1.44	9.206	8.536	0.379	0.443	0.032	0.030	0.851	0.746
48 h after operation	5.95±1.51	6.73±1.60	6.68±1.59	5.85±1.47	10.043	9.593	0.383	0.425	0.032	0.035	0.757	0.823
72 h after operation	3.32±1.58	4.99±1.72	4.67±1.67	4.03±1.69	6.283	7.115	0.847	0.912	0.039	0.036	0.402	0.390
Time												
Time to get out of bed	22.75±7.85	28.67±7.91	34.23±8.16	32.68±7.76	11.835	12.226	8.783	9.451	0.027	0.021	0.036	0.028
Time to extubation	98.41±12.03	123.38±11.92	146.43±11.97	132.14±11.69	19.768	15.565	10.423	8.724	0.026	0.09	0.035	0.020
Time to discharge	121.39±12.16	146.56±12.73	175.46±12.30	166.44±11.82	22.573	27.392	11.041	19.895	0.005	0.002	0.021	0.021
NRS: numeric rating scale.												

### 2.4 三组下床时间、拔管时间和出院时间比较

A组分别与B组、C组比较下床时间、拔管时间和出院时间, 以及B组与C组比较下床时间、拔管时间、出院时间, 差异均有统计学意义(*P* < 0.05)(表 3)。

## 3 讨论

ERAS理念由丹麦者Kehlet首次提出, 经由黎介寿院士和江志伟教授引进并推广。ERAS理念贯穿整个围手术期, 包含从入院的宣教开始, 术前准备、麻醉、手术、术后管理, 直至出院。术前准备方式的改进和手术术式的微创化均为ERAS中的重要内容^[6]^。

随着腔镜器械和技术的发展, 胸腔镜手术已成为肺癌手术主要的手术方式。单孔胸腔镜手术的出现和发展, 使得肺癌手术在胸腔镜手术的基础上进一步减少手术创伤^[7]^。本研究指出单孔手术可有效减轻术后疼痛。术后疼痛为手术常见的并发症, 部分患者可出现长期的疼痛, 严重影响患者的生活质量。术后疼痛是影响胸外科手术后快速康复的常见并发症, 术后疼痛减弱了患者术后深呼吸和咳嗽排痰, 从而增加了肺部感染、肺不张等呼吸道的并发症发生率^[8]^。胸腔镜手术时可能会损伤肋骨上下缘分布的神经, 肋间神经及其分支的卡压是引起切口疼痛的关键因素, 单孔手术的切口一般位于腋前线第4或5肋间, 其宽度一般较腋中线第7或8肋间要宽, 术中不需要使用Trokar, 而且术后经切口间隙留置胸管引流。多孔手术一般是在腋中线第7或8肋的观察孔置管, 另需做2个操作孔, 后胸壁行手术切口时, 因肋间隙较窄, 术中损伤肋间神经或副神经的风险增加, 后胸壁肌肉组织丰富, 切口时损伤较大^[9]^。单孔胸腔镜切口少, 避免后胸壁的切口, 损伤神经可能性小; 无Trokar的卡压, 以及切口肋间隙宽度大会减少对肋间神经及其分支的侵扰, 所以单孔胸腔镜有效减轻术后疼痛, 可提高术后生活质量, 也有利于患者尽快康复^[10]^。

肺部感染是胸外科术后发病率最高的并发症, 并可能引起其他并发症, 增加患者痛苦, 延长住院时间, 影响患者的快速康复^[11]^。Andalib等^[12]^发现肺癌术后未发生肺部感染的患者其5年生存率显著高于发生肺部感染的患者, 控制围手术期的肺部并发症不但可以促进患者的快速康复, 而且可以达到良好的远期预后。术前肺功能差、术中高流量通气造成的肺损伤均与术后发生肺部感染相关^[13]^。手术创伤和术中应激引起的炎症反应会增加机体对中枢和外周疼痛敏感性, 会提高胸膜和骨膜对疼痛介质的反应性, 从而产生明显的疼痛, 影响患者早期下床活动^[14]^。

快速康复理念指导下的呼吸功能锻炼可以有效提高肺功能, 增加呼吸肌, 尤其是膈肌、腹壁肌等位于腹部的呼吸肌的力量, 提升胸腹部呼吸肌的整体协调性, 有利于术后的有效咳嗽和排痰。有研究^[15]^指出术前行呼吸功能锻炼可有效降低呼吸指数和肿瘤坏死因子-α(tumor necrosis factor-α, TNF-α)水平, 提高术后氧合指数, 减轻术后的炎症反应, 提示通过呼吸功能锻炼可有效减轻肺损伤。罗洞波等^[16]^指出单孔胸腔镜手术较相比3孔胸腔镜手术, 炎症指标明显降低, 提示单孔胸腔镜手术可有效减轻术后炎症反应。单孔胸腔镜手术减轻术后疼痛, 呼吸功能锻炼改善肺功能, 有利于患者早期下床活动^[17]^。早期的下床活动和有效的咳嗽排痰, 使肺泡充分膨开, 各级支气管分泌物及时排出, 能防止肺不张的发生^[18]^。本研究结果显示, 经过短期的肺康复训练, 患者下床时间提前, 术后肺部感染发生率明显降低。

单孔胸腔肺癌手术与多孔肺癌手术比较, 在围手术期出血量、淋巴结清扫数目、手术时间、术后引流量方面无统计学差异, 提示两种手术方式对肺癌的治疗手术效果无明显差异, 但两组胸管留置时间有明显差异, 考虑与单孔手术可以有效减轻疼痛及早期的下床活动使肺充分复张将胸腔积液挤出胸腔有关^[19]^。在任占良等^[20]^的研究中单孔胸腔镜肺癌手术中ERAS组较对照组能显著缩短胸管拔管时间和住院时间, 其结果与本研究相符, 同时尽早拔除胸管可以进一步减轻患者的疼痛, 有利于患者的快速康复。

综上所述, 快速康复理念指导下的呼吸功能锻炼联合单孔胸腔镜手术能够减轻患者的疼痛, 减少肺癌手术患者的术后并发症, 缩短出院时间, 促进快速康复, 值得推广。

## References

[1] Ferlay J, Shin H, Bray F (2010). Estimates of worldwide burden of cancer in 2008: GLOBOCAN 2008. Int J Cancer.

[2] Chen W, Zheng R, Baade PD (2016). Cancer statistics in China, 2015. CA Cancer J Clin.

[3] Li CW, Xu MQ, Xu GW (2018). A comparative study of acute and chronic pain between single port and triple port video-assisted thoracic surgery for lung cancer. Zhongguo Fei Ai Za Zhi.

[4] Su JH, Yu PM, Zhou YB (2014). Influencing factor of postoperation fast-track recovery and in hospital cost after lobctomy for lung cancer. Zhongguo Fei Ai Za Zhi.

[5] Brunelli C, Zecca E, Martini C (2010). Comparison of numerical and verbal rating scales to measure pain exacerbations in patients with chronic cancer pain. Health Qual Life Outcomes.

[6] Gao SS, Li LY, Shi QT (2018). Application of enhanced recovery after surgery concept in the perioperative care of whole endoscopic esophageal cancer surgery. Zhonghua Xiong Bu Wai Ke Dian Zi Za Zhi.

[7] Sihoe AD (2016). Uniportal video-assisted thoracic (VATS) lobectomy. Ann Cardiothorac Surg.

[8] Xiang R, Xie TP, Yang XJ (2015). Clinical effect of incision analgesia pump after open lobectomy. Zhonghua Xiong Xin Xue Guan Wai Ke Za Zhi.

[9] Lin Y, Zheng W, Zhu Y (2016). Comparison of treatment outcomes between single-port video-assisted thoracoscopic anatomic segmentectomy and lobectomy for non-small cell lung cancer of early-stage: a retrospective observational study. J Thorac Dis.

[10] Wu XY, Zhang M, Wang ZH (2019). Multi-factor analysis of postoperative pain after single-port thoracoscopic radical resection of lung cancer. Zhongguo Wei Chuang Wai Ke Za Zhi.

[11] Zhou K, Su JH, Lai YT (2017). Effect of preoperative pulmonary rehabilitation on postoperative pneumonia in patients with lung cancer. Zhonghua Xiong Bu Wai Ke Dian Zi Za Zhi.

[12] Andalib A, Ramana-Kumar AV, Bartlett U (2013). Influence of postoperative infectious complications onlong-term survival of lung cancer patients: a population-based cohort study. J Thorac Oncol.

[13] Urba SG, Orringer MB, Turrisi A (2001). Randomized trial of preoperative chemoradiation therapy versus surgery alone in patients with locoregional esophageal carcinoma. Clin Oncol.

[14] Wang ZH, Tao S, Tan XJ (2019). Effect of target exercise on rapid perioperative recovery of patients undergoing single-hole thoracoscopic lobectomy. Zhonghua Fei Bu Ji Bing Za Zhi (Dian Zi Ban).

[15] Shi QT, Li LY, Xu QQ (2019). Application of rapid rehabilitation concept in reducing postoperative pulmonary infection in patients with total laparoscopic esophageal cancer. Zhonghua Xiong Bu Wai Ke Dian Zi Za Zhi.

[16] Luo DB, Gao YF, Wu ZH (2017). Uniportal and the three ports thoracoscope contrast on immune function effects in patients with lung cancer. Lin Chuang He Shi Yan Yi Xue Za Zhi.

[17] Sokol G, Vilozni D, Hakimi R (2015). The short-term effect of breathing tasks via an incentive spirometer on lung function compared with autogenic drainage in subjects with cystic fibrosis. Respir Care.

[18] Tao S, Wang ZH, Gu JL (2019). Analysis of the effect of rapid rehabilitation surgical nursing on lung function of patients after single-hole thoracoscopic lobectomy. Zhonghua Fei Bu Ji Bing Za Zhi (Dian Zi Ban).

[19] Li W, Zheng ZF, Zhuang HQ (2019). Comparative analysis of clinical effects of single-hole and three-hole thoracoscopic radical lung cancer surgery. Dang Dai Yi Xue.

[20] Ren ZL, Zhang Y, Ren XP (2017). Application of enhanced recovery after surgery in single port thoracoscopic radical resection of lung cancer. Zhonghua Qiang Jing Wai Ke Za Zhi (Dian Zi Ban).

